# The Impossible Leffingwell

**Published:** 1899-05

**Authors:** 


**Monthly Review of Medicine and Surgery.**

*EDITORS:*

THOMAS LOTHROP, M. D. - WM. WARREN POTTER, M. D.

All communications, whether of a literary or business nature, should be addressed to the managing editor: 284 FRANKLIN STREET, BUFFALO, N. Y.

VOL. XXXVIII. MAY, 1899. NO. 10.

### THE IMPOSSIBLE LEFFINGWELL.

SENATOR GALLINGER recently fathered a bill prohibiting animal experimentation in the District of Columbia, and in doing so was the means of turning loose upon the public one of the most insufferable of the narrow-minded and vituperative anti-vivisection cranks that it has been the misfortune of the medical profession to turn out. Heaven only knows what high motive could inspire a member of the Senate to father such a bill—a senate notorious for its vivisection of trade and humanity—but it did it.

They do queer things in the senate anyway. It is a sort of a political crazy quilt these days, with patches from the beef embalming works and other patches from the monopoly circles. Senator Gallinger climbed into the ring with his anti-vivisection bill, and he was supported by a New York State doctor named Leffingwell, who flew to Gallinger's aid in his effort to bar the progress of conservative research, like a hungry vulture to a carrion feast. He labored and brought forth from the recesses of his mind as disgraceful an attack as was ever issued by anti-vivisectionists, who are notorious for the falsity, malice and bigotry of their arguments.

This man Leffingwell prepared an argument for Gallinger; an argument in pamphlet form in which he endeavored to show the awful cruelties of vivisection. How he must have gloated over the opportunity to throw himself into the rays of the lime light!

After properly prefacing his work, Leffingwell opens his argument with an attack on a Catholic order, and in the bitterness of this is shown his bigotry. After accusing the members of this order of being chronic liars, he says on his own responsibility and with never a blush of shame that on a recent visit to the Pasteur Institute in Paris, he saw "scores of rabbits lying in their compartments slowly dying, *with their eyes rotting out* (italics his), the result of inoculations which the American Academy of Sciences informs Congress 'involved less suffering than the administration of an anesthetic.'"

Leffingwell is certainly an impossibility. Anyone is who would attack a religious order for lying and then make such a statement as that.

Then this eminent anti-vivisectionist proceeds to an attack on Surgeon General Sternberg. We do not know what his grievance against the Surgeon General of the Army is. He must have one, else he is a malicious slanderer. If he has a grievance it is a selfish one.

After attacking General Sternberg as one of the cruel experimenters, Leffingwell gives an account of certain experiments made by the Surgeon General so worded as to make Dr. Sternberg appear to be a most degraded and loathsome person. If there ever was any doubt regarding the malice of Leffingwell, this section of his pamphlet would clear it up.

When the pamphlet appeared, Dr. Sternberg replied to certain portions of it in a letter addressed to the chairman of the committee on the District of Columbia, in which he said:

The fact is recognised by well-informed physicians throughout the world that the great progress made by scientific medicine during the past thirty years has been to a great extent due to experiments upon the lower animals, and that our exact knowledge with reference to the cause and prevention of infectious diseases of man and domestic animals is based largely upon the inoculation experiments, made for the most part upon small animals, such as mice, guinea-pigs and rabbits. Those who have engaged in such experiments insist that the pain attending the inoculation is trifling and does not call for the administration of an anesthetic.

To give an anesthetic to an inoculated animal during the time it is under observation to determine the result of the experiment would entirely neutralise the value of the experiment, and a law requiring this would effectually arrest all investigations of this kind. Dr. Leffingwell has referred to certain experiments upon rabbits made by me in the laboratory of the Johns Hopkins University nearly twenty years ago. Those experiments resulted in a very important scientific discovery—that of the germ of pneumonia. If Dr. Leffingwell had consulted a modern text-book of bacteriology he would have found that in the experiments referred to the fatal result in the rabbits experimented upon was due to the accidental presence in my salivary secretions of a pathogenic micrococcus, which has since been proved to be the usual cause of inflammation of the lungs, also that it has since been found temporarily present in the salivary secretions of many other healthy individuals as well as in the pulmonary secretions of persons suffering from pneumonia. Dr. Leffingwell's incomplete account of my experiments, and his incorrect statement as to the cause of the death of the rabbits experimented upon, have been published in the daily newspapers with a view to presenting me to the public as a man of cruel instincts, and also as one who carried in his mouth 'a venom more deadly than that of the viper or the rattlesnake.' Both allegations are false. If I had hunted rabbits with dogs there might have been some foundation for the charge of cruelty. But no one has proposed that the Congress of the United States shall legislate to prevent the wounding and killing of animals for sport, while my experiments made in the interest of science and humanity are characterised by Dr. Leffingwell and his associates in this crusade against scientific investigation as cruel, and an effort is being made to convince the Senate of the United States that the government laboratories in this city should be placed under surveillance other than that of the bureau officers who, under present laws and regulations, are in charge of them.

This is a manly, straightforward reply to a malicious attack and is what might be expected of the Surgeon General.

Dr. Sternberg occupies a prominent place in the public service and is professionally, one of the foremost bacteriologists in the world. Under the circumstances he is the target for all the disgruntled and mentally distorted backbiters in and out of the profession. It is regretable, however, that the Surgeon General should be the victim of these attacks. Praise alone should be his, if not for his eminent scientific work, then for his admirable conduct of the medical department of the army during the war. Of all the bureaus his is the safest from attack, political or otherwise. He has furnished the best medical attendance obtainable, and the drugs and supplies sent out by his order have not been "as good as any," but have been unqualifiedly the best. A man who has done what Gen. Sternberg has done can withstand the attacks of such as Leffingwell without injury.

Truly, Leffingwell is an impossibility.

